# Editorial for Special Issue: “Supramolecular Nanomaterials for Biomedical Application”

**DOI:** 10.3390/nano13061054

**Published:** 2023-03-15

**Authors:** Sa Wang, Minhuan Lan, Huiqing Peng, Jinfeng Zhang

**Affiliations:** 1 Key Laboratory of Molecular Medicine and Biotherapy, School of Life Sciences, Beijing Institute of Technology, Beijing 100081, China; 18340876735@163.com; 2 College of Chemistry and Chemical Engineering, Central South University, Changsha 410083, China; 3 Beijing Advanced Innovation Center for Soft Matter Science and Engineering, State Key Laboratory of Chemical Resource Engineering, Beijing University of Chemical Technology, Beijing 100029, China

Since the discovery of supramolecular chemistry in 1987, the field has attracted great attention from researchers in chemistry, materials science, and other fields. Supramolecular nanomaterials usually refer to the synthesis of materials with diverse structures and functions by the self-assembly of simple molecular entities through noncovalent interactions including hydrogen bonding and Van der Waals forces, as well as electrostatic, hydrophobic, π–π, or host–guest interactions. In the past decades, a wide variety of self-assembled building blocks, such as small organic molecules (e.g., drug molecules and fluorescent dyes), semiconducting polymers, dendrimers, and biomacromolecules (e.g., peptides, nucleic acids, or proteins) have been extensively employed for constructing supramolecular nanomaterials [1,2,3,4,5]. More importantly, owing to their synthetic flexibility, stimuli responsiveness, excellent biocompatibility, improved therapeutic performances, and reduced side effects, supramolecular nanomaterials have been considered to be one of the most promising candidates for various biomedical applications such as imaging, biosensing, drug carrying, and disease treatments [6,7,8,9,10].

Due to the remarkable potential of supramolecular nanomaterials in the field of biomedicine, many innovative works regarding these have emerged in recent years, covering topics from basic research to practical utilization. This Special Issue, “Supramolecular Nanomaterials for Biomedical Application”, aims to introduce the design strategy, advanced functionality, and biomedical applications of supramolecular nanomaterials. In particular, this Special Issue includes five research papers (three articles and two reviews) covering the preparation, characterization, and biomedical application of supramolecular nanomaterials.

Zhu et al. synthesized water-dispersible glutathione-modified CdTe quantum dots (GSH-CdTe QDs) by covalently bonding Cd atoms on the surface of CdTe QDs and thiol groups of the GSH, which further reacted with the Ru (II) complex via electrostatic adsorption to synthesize the QDs-Ru complexes to achieve the rapid and sensitive identification of plasma cell-free DNA (cfDNA) biomarkers in cancer patients [11]. Considering that graphene-based nanomaterials can facilitate the process of neurogenesis in vitro, using different microscopy techniques and real-time gene-expression analysis, Simonovic et al. explored the ability of liquid-phase-exfoliated graphene films to induce and stimulate the neural differentiation of stem cells from apical papilla (SCAP) [12]. Lee et al. utilized simple and scalable top-down and bottom-up approaches to prepare reduced-graphene quantum dots (RGQDs) and hyaluronic acid–graphene quantum dots (HGQDs), which possessed substantial near-infrared (NIR) absorption and fluorescence throughout the visible and NIR ranges, showing significant photothermal performance as well as NIR and fluorescence-imaging capabilities in HeLa cells [13]. Bahutair et al. reviewed the recent applications of ultrasound-stimulated drug release from liposomal systems, stated the various factors affecting the sonosensitivity of liposomes and the mechanisms of ultrasound-stimulated drug release, and finally summarized the studies of ultrasound-induced liposome smart-drug-delivery systems (SDDSs) in vitro and in vivo [14]. Abed et al. summarized the chemical composition and design strategies of redox-responsive drug-delivery systems applied to anticancer treatments, focusing on some major redox-responsive chemical groups. In addition, they explored disulfide-containing liposomes, polymeric micelles, and nanogels as carriers [15].

This Special Issue describes the preparation, characterization, and biological application of supramolecular nanomaterials. We expect it to provide useful guidance for the further development of efficient and multifunctional supramolecular nanomaterials for biomedical applications.

## References

[1] Zhang K., Gao Y.-J., Yang P.-P., Qi G.-B., Zhang J.-P., Wang L., Wang H. (2018). Self-Assembled Fluorescent Organic Nanomaterials for Biomedical Imaging. Adv. Healthc. Mater..

[2] Xing P., Zhao Y. (2016). Multifunctional Nanoparticles Self-Assembled from Small Organic Building Blocks for Biomedicine. Adv. Mater..

[3] Cui X., Lu G., Dong S., Li S., Xiao Y., Zhang J., Liu Y., Meng X., Li F., Lee C.-S. (2021). Stable π-radical nanoparticles as versatile photosensitizers for effective hypoxia-overcoming photodynamic therapy. Mater. Horizons.

[4] Peng H.-Q., Liu B., Wei P., Zhang P., Zhang H., Zhang J., Li K., Li Y., Cheng Y., Lam J.W.Y. (2019). Visualizing the Initial Step of Self-Assembly and the Phase Transition by Stereogenic Amphiphiles with Aggregation-Induced Emission. ACS Nano.

[5] Huang L., Zhao S., Fang F., Xu T., Lan M., Zhang J. (2021). Advances and perspectives in carrier-free nanodrugs for cancer chemo-monotherapy and combination therapy. Biomaterials.

[6] Zhu L., Luo M., Zhang Y., Fang F., Li M., An F., Zhao D., Zhang J. (2023). Free radical as a double-edged sword in disease: Deriving strategic opportunities for nanotherapeutics. Coord. Chem. Rev..

[7] Fang F., Li M., Zhang J., Lee C.S. (2021). Different Strategies for Organic Nanoparticle Preparation in Biomedicine. ACS Mater. Lett..

[8] Zhu W., Li Y., Guo S., Guo W.-J., Peng T., Li H., Liu B., Peng H.-Q., Tang B.Z. (2022). Stereoisomeric engineering of aggregation-induced emission photosensitizers towards fungal killing. Nat. Commun..

[9] Fang F., Zhu L., Li M., Song Y., Sun M., Zhao D., Zhang J. (2021). Thermally Activated Delayed Fluorescence Material: An Emerging Class of Metal-Free Luminophores for Biomedical Applications. Adv. Sci..

[10] Zhang J., Nie W., Chen R., Chelora J., Wan Y., Cui X., Zhang X., Zhang W., Chen X., Xie H.-Y. (2019). Green Mass Production of Pure Nanodrugs via an Ice-Template-Assisted Strategy. Nano Lett..

[11] Zhu L., Zhao D., Xu L., Sun M., Song Y., Liu M., Li M., Zhang J. (2022). A Fluorescent ‘‘Turn-On’’ Clutch Probe for Plasma Cell-Free DNA Identification from Lung Cancer Patients. Nanomaterials.

[12] Simonovic J., Toljic B., Lazarevic M., Markovic M.M., Peric M., Vujin J., Panajotovic R., Milasin J. (2022). The Effect of Liquid-Phase Exfoliated Graphene Film on Neurodifferentiation of Stem Cells from Apical Papilla. Nanomaterials.

[13] Lee B., Stokes G.A., Valimukhametova A., Nguyen S., Gonzalez-Rodriguez R., Bhaloo A., Coffer J., Naumov A.V. (2023). Automated Approach to In Vitro Image-Guided Photothermal Therapy with Top-Down and Bottom-Up-Synthesized Graphene Quantum Dots. Nanomaterials.

[14] Bahutair W.N., Abuwatfa W.H., Husseini G.A. (2022). Ultrasound Triggering of Liposomal Nanodrugs for Cancer Therapy: A Review. Nanomaterials.

[15] Abed H.F., Abuwatfa W.H., Husseini G.A. (2022). Redox-Responsive Drug Delivery Systems: A Chemical Perspective. Nanomaterials.

